# Contribution of Gut Microbiota-Derived Uremic Toxins to the Cardiovascular System Mineralization

**DOI:** 10.3390/toxins13040274

**Published:** 2021-04-10

**Authors:** Iwona Filipska, Agata Winiarska, Monika Knysak, Tomasz Stompór

**Affiliations:** Department of Nephrology, Hypertension and Internal Medicine, University of Warmia and Mazury in Olsztyn, 10-561 Olsztyn, Poland; filipskaiwona@gmail.com (I.F.); agatawiniarska@hotmail.com (A.W.); makb@poczta.fm (M.K.)

**Keywords:** chronic kidney disease (CKD), vascular calcification, mineral and bone disorders of CKD, p-cresyl sulphate, indoxyl sulphate, trimethylamine-*N*-oxide, phosphate

## Abstract

Chronic kidney disease (CKD) affects more than 10% of the world population and leads to excess morbidity and mortality (with cardiovascular disease as a leading cause of death). Vascular calcification (VC) is a phenomenon of disseminated deposition of mineral content within the media layer of arteries preceded by phenotypic changes in vascular smooth muscle cells (VSMC) and/or accumulation of mineral content within the atherosclerotic lesions. Medial VC results in vascular stiffness and significantly contributes to increased cardio-vascular (CV) morbidity, whereas VC of plaques may rather increase their stability. Mineral and bone disorders of CKD (CKD-MBD) contribute to VC, which is further aggravated by accumulation of uremic toxins. Both CKD-MBD and uremic toxin accumulation affect not only patients with advanced CKD (glomerular filtration rate (GFR) less than 15 mL/min/1.72 m^2^, end-stage kidney disease) but also those on earlier stages of a disease. The key uremic toxins that contribute to VC, i.e., p-cresyl sulphate (PCS), indoxyl sulphate (IS) and trimethylamine-*N*-oxide (TMAO) originate from bacterial metabolism of gut microbiota. All mentioned toxins promote VC by several mechanisms, including: Transdifferentiation and apoptosis of VSMC, dysfunction of endothelial cells, oxidative stress, interaction with local renin–angiotensin–aldosterone system or miRNA profile modification. Several attractive methods of gut microbiota manipulations have been proposed in order to modify their metabolism and to limit vascular damage (and VC) triggered by uremic toxins. Unfortunately, to date no such method was demonstrated to be effective at the level of “hard” patient-oriented or even clinically relevant surrogate endpoints.

## 1. Introduction: Pathologic Calcification in Cardiovascular System

Vascular calcification (VC) is defined as a process of organized hydroxyapatite crystal deposition within blood vessel walls. To some extent it can be considered physiologic (as an element of physiologic aging, when calcification is limited to the atherosclerotic plaques), but in certain chronic diseases, such as: Type 1 or type 2 diabetes, metabolic syndrome, chronic kidney disease (CKD) or postmenopausal osteoporosis, it may be excessive and may contribute to the cardiovascular disease and, in consequence, to increased cardiovascular (CV) mortality. VC is a well-recognized and confirmed risk factor for CV and cerebrovascular events and mortality resulting from these events [1]. VC can affect both intima and media layers of the arteries. When it affects intima, it is usually limited to the atherosclerotic plaque (and is seen as a “patchy” calcification on imaging) and may contribute to the arterial lumen narrowing (although several data suggest that calcification may also be considered as a process that stabilizes the plaque and may protect against its rupture) [2]. Calcification of media (also known as Mönckeberg sclerosis) is more generalized and disseminated (seen as “linear” upon imaging). It leads to arterial stiffness, contributes to systolic hypertension with increased pulse pressure, promotes the development of heart failure with preserved ejection fraction and increases the risk of stroke and myocardial infarction (but in the mechanism distinct from plaque instability and rupture) [3]. VC progresses along with decline in glomerular filtration rate (GFR) and is most advanced in end-stage kidney disease (ESRD) patients treated with dialysis [4]. We were the first who described the prevalence, advancement and progression of coronary artery calcification in patients treated with peritoneal dialysis [5,6]. Adeney et al. demonstrated that even in patients with moderately advanced CKD (eGFR < 60 mL/min per 1.73 m^2^, mean 50.6 mL/min per 1.73 m^2^) Shanahan without symptomatic cardiovascular disease (CVD), but with high serum phosphate level the prevalence and advancement of coronary artery calcification (CAC), calcification of descending aorta and mitral valve were significantly more pronounced as compared to subjects with normal kidney function [7]. Several studies reported the association between the risk of CV events and CV death and the degree of VC identified in several locations (coronary arteries, aortic arch and abdominal aorta) in different CKD patient groups (peritoneal dialysis, hemodialysis and pre-dialysis) [8,9,10]. The risk of cardiovascular and cerebrovascular events attributed to VC is largely independent from “traditional” risk factors for development of atherosclerosis, such as smoking, obesity or serum LDL-cholesterol [11,12,13,14]. “Non-traditional” risk factors present exclusively in CKD patients or are at least much more pronounced as compared to other patient groups include gut microbiota-derived uremic toxins.

## 2. Selected Mechanisms of VC Regulation

Calcium and phosphate metabolism abnormalities (at the clinical level known as mineral and bone disorders of chronic kidney disease (CKD-MBD)) are essential in the initiation and then maintenance of VC [15]. VC is an active, precisely regulated process, which resembles the bone formation [16]. Its activators include (among others): Calcium and phosphate (that comprise not only substrates to form hydroxyapatite but also serve as active regulators of own deposition), several proinflammatory cytokines, oxidative stress and—when considering CKD—uremic toxins [16,17,18,19]. Traditionally uremic toxins are considered as substances accumulating in advanced CKD (eGFR < 15 mL/min/1.73 m^2^ and/or symptomatic uremia). It must be noted however that they start to accumulate early in the course of CKD and may predict cardiovascular events across all stages of CKD [20,21].

It seems that endothelial dysfunction and phenotypic changes within vascular smooth muscle cells (VSMC) are the key factors facilitating VC at the cellular level. VSMC comprise the key cellular component of VC. They play crucial role in maintaining the normal structure and function of vessels—apart from their contractile function that contributes to the fine adjustment of vascular resistance, vascular compliance and blood pressure regulation they also synthetize extracellular matrix (ECM), essential to maintain the normal structure of the vessels. Under pathological conditions the latter ability becomes dysregulated and paves the way to vascular remodeling [22]. Function of VSMC can be modified by inorganic phosphate (Pi)—in hyperphosphatemia VSMC uptake an excess of Pi, which may induce their transdifferentiation into osteoblast-like cells or trigger apoptosis, with release of extracellular vesicles (resembling osteoblastic vesicles found in the bone undergoing the processes remodeling and mineralization). Pit-1 facilitates transmembrane transport of Pi into VSMC and genetic (gene knock-out) or pharmacological manipulations on this transport can inhibit VC [23]. Thus, VSMC create a microenvironment for pathologic VC.

Following phosphate absorption VSMC transdifferentiate into osteoblast-like cells, loose their ability to synthetize and secrete inhibitors of calcification, and—in turn—activate the repertoire of genes encoding proteins that contribute to hydroxyapatite deposition within the media layer. This process is accompanied with ECM remodeling and release of extracellular vesicles (including exosomes and microvesicles). Extracellular vesicles are depleted with inhibitors of calcification (such as, for example fetuin A or matrix Gla protein) and may serve as “nuclei of calcification”. The same role may also be assigned to VSMC that undergo apoptosis [24]. Endothelial cells also contribute to VC. Microvesicles released from endothelial cells upon treatment with indoxyl sulphate (IS) and p-cresyl sulphate (PCS) contain the repertoire of miRNA with the potential to induce apoptosis and senescence of other endothelial cells, impair their regenerative capacity and to activate inflammation [25,26]. IS-induced, endothelial cell-derived microvesicles, when cultivated with VSMC, induced the expression of genes that encode calcification-promoting proteins and stimulated in vitro calcification in the latter cell line [27]. The IS-induced cross-talk between endothelial cells and VSMC may also be mediated by cytokines. Bouabdallah et al. demonstrated that supernatant from vascular endothelial cells treated in culture with indoxyl sulphate induced VSMC calcification in vitro. The supernatant analysis allowed for identification of interleukin 8 as a mediator of such an induction [28].

Uremic toxins critically contribute to the initiation and then—progression of VC. The most important substances identified to impact on the endothelial cells and VSMC, and to promote VC include: Advanced glycation end-products, PCS, IS and trimethylamine-*N*-oxide (TMAO); Pi and phosphaturic hormones (such as parathyroid hormone and phosphatonin FGF23 are also classified by some authors as uremic toxins) [29,30,31]. Since gut microbiota is the most important source of PCS, IS and TMAO, uremia-dependent or kidney disease-specific gut microbiota abnormalities (dysbiosis) may also contribute to VC [32,33].

Epigenetic regulation by miRNA is an increasingly recognized mechanism that controls many biological processes, including pathologic calcification. miRNA are small (20–25 nucleotides) non-coding RNA particles that modify the expression of target genes via promotion or inhibition of degradation of respective mRNA, based on their complementarity [34]. Given their crucial impact on gene expression now they are considered as new disease biomarkers and potential therapeutic targets or even therapeutic agents. In case of pathologic calcification Massy et al. have demonstrated in mouse models that multiple miRNAs (such as: miR-126, miR-143, miR-145 and miR-223) as well as their target genes (and in consequence—proteins) become modified in the course of CKD and atherogenesis. Cellular expression of particular miRNA was correlated with such classical biomarkers of CKD and/or atherosclerosis, as: Serum cholesterol, urea or calcium. Interestingly, the similar changes in miRNA profile within VSMC and endothelial cells were found in pre-dialysis CKD patients, patients on hemodialysis and renal transplant recipients. The latter finding might confirm the role of particular set of miRNA as a “fingerprint” of vascular lesions attributable to CKD [35].

## 3. Gut Microbiota as a Source of Uremic Toxins. Impact of CKD on the Gut Microbiota

There are 100 trillion (10^18^) bacteria which reside within the gut of healthy humans and the number of their genes (ca. 3 million) exceed several times the number of the human host genes [36,37,38]. These high numbers result in enormous although not fully recognized and understood impact on the host metabolism and physiology, with special significance for immune system [39].

Bidirectional relationship exists between CKD and gut microbiota: Bacterial turnover of ingested nutrients leads to generation of toxic substances that are absorbed and accumulate along with the loss of renal function, but CKD has also potential to modify the composition of gut microbiota. This relationship can be considered a vicious circle since uremic toxins generated by microbiota possess certain nephrotoxic potential aggravating CKD, further modifying microbiota composition and enhancing toxin accumulation [40]. The gut microbiota significantly contributes to the development of uremic symptoms and long-term consequences of CKD (and to the progression of CKD), but also plays an important role on very early stages of CKD (or even as a factor that initiates kidney injury). For example, several gut-derived uremic toxins have been demonstrated to induce podocyte injury or to stimulate kidney fibrosis [41,42,43,44,45,46,47].

The toxins that are the subjects of the present review are derived from the metabolism of dietary tryptophan (IS), phenylalanine and tyrosine (PCS) and choline, phosphatidylcholine and L-carnitine (TMAO) [45]. Phenylalanine and tyrosine are converted to 4-hydroxyphenylacetic acid and then decarboxylated by p-hydroxyphenylacetate decarboxylase into p-cresol. p-cresol is further metabolized by cytosolic sulfotransferase to form PCS while passaging across the gut-blood barrier. Tryptophan is first converted to indole by tryptophan indol-lyase, and indole turns into IS in the liver. Finally, TMA is synthetized from choline and phosphatidylcholine by choline TMA-lyase and from carnitine—by carnitine oxygenase; further transformation into TMAO is facilitated by the hepatic flavin monooxygenae 3 [41,48,49].

Urea and other toxins accumulating in CKD reach the GI lumen and impact on microbiota composition. The detailed discussion on particular species that increase or decrease under the setting of CKD is beyond the scope of this review. Nonetheless, the observed changes may be summarized in the following way: The amount of bacteria equipped with urease, uricase and the above mentioned enzymes that participate in tryptophan, phenylalanine, tyrosine, choline, phosphatidylcholine and L-carnitine turnover increase, whereas population of species able to synthesize short-chain fatty acids (such as: Acetate, butyrate, propionate and D-lactate) decreases. The loss of a potential to synthetize short-chain fatty acids (especially butyrate) within the gut lumen exposes CKD patients to the additional health risks [39,44,45,46,50,51]. Distinct microbiological profiles of microbiota have been described for CKD in general, but also for particular kidney diseases, such as: Diabetic kidney disease, lupus nephritis and primary glomerular diseases (for example IgA nephropathy) [32].

The changes in microbiota composition in CKD patients largely depend on the substantial changes in diet composition and intake of nutrients due to restrictions recommended in order to prevent CKD progression and to avoid certain complications of CKD (including VC). Although the benefits of fruits and vegetables (F&V) were also demonstrated in CKD patients (plant proteins contain less phosphate, plant-derived phosphate is less absorbable, F&V have potential for alkalization which slows down the progression of CKD, protects from CKD-MBD and prevents muscle loss), such a diet can rarely be consumed due to the threat of hyperkalemia [52,53]. It should be emphasized that many drugs necessary to treat CKD and its complications (such as: Angiotensin converting enzyme inhibitors, angiotensin II receptor blockers, beta-blockers, mineralocorticosteroid receptor antagonists, heparins and calcineurin inhibitors) have a potential to increase potassium. In order to compensate for drug-associated hyperkalemia (especially when the particular drug should not be discontinued) low potassium diet must be recommended. This is one of the reasons why the patients with extremely high cardiovascular risk cannot eat healthy diets (such as Mediterranean or Dietary Approach to Stop Hypertension [DASH] diets) [54,55]. Drugs use for the treatment of CKD and its sequelae may also modify the composition of gut microflora [39]. Infection remains the second most frequent complication and cause of hospitalization among CKD patients (preceded only by cardiovascular disease), hence the use of broad spectrum antibiotics, substantially influencing the gut microbiota is very high in this patient group [56,57,58]. The burden of infection and common use of antibiotics is also illustrated by very high rate of antibiotic resistance as well as high risk of Clostridium difficile infection in CKD [59,60]. The use of phosphate binders, overuse of proton-pump inhibitors, and iron supplementation prescribed to treat renal anemia possess another challenges to normal composition of gut microbiota in CKD [61,62,63].

Increased synthesis of uremic toxins in a gut lumen is accompanied by their increased bioavailability due to a “leaky gut” syndrome. Chronic inflammation is a hallmark of CKD; it involves most body systems (including gastrointestinal tract) and results in translocation of endotoxins, bacterial fragments and uremic toxins across the GI mucosa into the bloodstream. This phenomenon promotes injury of several tissues and organs, including vessels, and contributes to endothelial dysfunction, vascular damage and VC [64]. Endotoxins, bacterial fragments and uremic toxins leaking from GI into bloodstream additionally fuel the state of chronic inflammation, which exemplifies another aspect of vicious circle in CKD [39].

In the following sections we aimed to describe the role of gut-derived uremic toxins in vascular injury and calcification.

## 4. Uremic Toxins Involved in the Vascular Calcification

### 4.1. Indoxyl Sulphate (IS)

IS belongs to low molecular weight, protein-bound toxins accumulating along with decreasing GFR; in ESRD patients treated with maintenance hemodialysis only unbound (free) fraction of IS is cleared during this procedure. Among several mechanisms of toxicity IS contributes to the development and progression of cardiovascular disease. The ability of IS to promote VC is one of the key mechanisms of cardiovascular damage attributed to this toxin. The promotion of VC triggered by IS has been described in a number of in vivo and in vitro experiments [65]. IS—among other mechanisms—induces VC via an enhancement of the oxidative stress. IS and PCS upregulate the expression of master transcription factor regulating inflammation (i.e., nuclear factor-kappa B (NF-κB)) and downregulate the master transcription factor that governs anti-oxidative defense (i.e., nuclear erythroid 2-related factor 2 (Nrf2)) [66]. IS may also promote VC on an epigenetic pathway. It has been demonstrated that miRNAs regulate osteoblastic VSMC transdifferentiation and IS may contribute to this mechanism [67]. miR-29b suppresses Wingless-related integration site (Wnt)/β-catenin pathway, a well-recognized trigger of VC, thus exerting protective role against VC. Zhang and colleagues demonstrated that miR-29b is downregulated in radial arteries from ESRD patients as well as in human aortic VSMC and that such a downregulation is triggered by IS [68]. Nakano et al. found in a mouse model of CKD that IS can accelerate atherogenesis and VC by stimulation of proinflammatory (M1) macrophages [65]. In rats with adenosine-induced kidney injury Opdebeeck et al. have shown that both IS and PCS induce VC, when achieving serum concentrations corresponding with those found in patients with CKD [69].

Excess renin–angiotensin–aldosterone (RAA) axis activation and increased concentration of angiotensin II (AngII) are well recognized factors that contribute to endothelial cell and VSMC injury. AngII can directly induce VC since it can activate receptor activator for nuclear factor κB ligand (RANKL); type 1 receptors for ATII (AT1 receptors) seem to be crucial in mediating RAA and RANKL interaction [70]. The ability of IS and PCS to activate RAA axis, to upregulate AT1 receptors and downregulate AT2 receptors has been demonstrated in experimental models of chronic kidney injury; similar interaction can also be assumed in vascular damage, remodeling and possibly VC [43]. Shimizu et al. found that in CKD rats IS in concentrations corresponding with those found in CKD patients potentiates the detrimental effect of AngII on VSMC and that this effect largely depends on IS-induced oxidative stress [71]. Moreover, in their earlier study the same authors pointed onto the role of IS as a mediator that increases the sensitivity of VSMC to platelet-derived growth factor (PDGF) and that this phenomenon was also linked to oxidative stress induced by IS [71,72,73].

IS has been identified as one of the key mediators of cardiovascular system injury in CKD as a well-recognized endotheliotoxin. It promotes the proinflammatory phenotype of endothelial cells by means of NF-κB upregulation, reduces NO availability, stimulates endothelial stress, promotes thrombosis and oxidative stress. It also alters endothelial progenitor cell migration and proliferation. All these mechanisms may constitute a “primum movens” for both atherosclerosis and VC [74].

As we mentioned previously, VC critically depends on serum Pi concentration and Pit-1 activity, the transport system facilitating an influx of Pi into VSMC. Wu et al. demonstrated the dose-dependent increase of Pit-1 mRNA and protein in VSMC upon exposure to IS. IS also induced an increased expression of bone morphogenetic protein 2 (BMP-2) and osteoprotegerin as well as calcium content in VSMC [75].

### 4.2. p-Cresyl Sulphate (PCS)

PCS is another protein-bound (and thus poorly dialyzable) uremic toxin that has been linked to cardio-vascular system damage and cardio-vascular outcome in CKD patients. As in the case of IS, PCS also originates from the gut microbiota metabolism and increases in serum along with decreasing GFR. Opdebeeck et al. in their experiments in a rat model described the mechanisms of how both mentioned solutes induce cardiovascular system injury. Both IS and PCS stimulated calcification of the aorta and peripheral arteries. Calcium content in aortic wall following exposure to these toxins increased 10-fold as compared to control animals; significant increase was also observed in the carotid and femoral arteries. On cross-sections of calcified vessels excess calcification has been noticed, that involved predominantly a media layer. The degree of aortic calcification correlated with serum calcium, IS, PCS and glucose. Activation of inflammation (acute phase response) and coagulation pathways were also observed in the aortic wall upon exposition to IS and PCS. Interestingly, short term (4 days) exposure to IS and PCS was sufficient to activate all incremented pathways potentially involved in excess calcification; long-term exposure (7 weeks) resulted already in significant calcification [76].

Han et al. demonstrated that—as discussed above for IS—PCS can also promote migration and proliferation of VSMC, the key cellular events in the development of VC. In atherosclerosis-prone apolipoprotein A-deficient [ApoE (-/-)] mice fed with high fat diet exposure to PCS resulted in significantly more pronounced atherosclerotic plaque formation as compared to control animals. In addition, PCS led to imbalance between matrix metalloproteinases and tissue metalloproteinase inhibitors, thus contributing to plaque instability [77].

Gross et al. in their research focused on the possible contribution of PCS to oxidative stress. Indeed, the authors demonstrated that PCS activates oxidative stress both in endothelial cells and VSMC. In addition, PCS promoted phenylephrine-induced contraction of smooth muscles within aortic wall and in consequence—promoted aortic wall remodeling, largely resembling remodeling found in patients with chronic uremia. It should be emphasized that all the above results obtained in an animal model were triggered by PCS in concentrations corresponding to those found in patients with advanced CKD (i.e., GFR of less than 15 mL/min/1.73 m^2^ or treated with maintenance dialysis) [78].

### 4.3. Trimethylamine-N-Oxide (TMAO)

TMAO is another uremic toxin originating from gut microbiota metabolism [79]. The role of TMAO in the development of calcification as well as the mechanisms by which this toxin contributes to VC have not been fully elucidated. Zhang et al. have confirmed in rat model that TMAO induces VC via an induction of phenotypic transformation in VSMC. TMAO has been shown to increase calcium content within animal VSMC cultured in a calcifying milieu in a dose-dependent manner. TMAO induced mRNA expression of the genes encoding proteins involved in osteoblastic differentiation of VSMC, including Runt-related transcription factor 2 (Runx2) and bone morphogenetic protein 2 (BMP2). The above observations were further confirmed in cultured human VSMC. The same phenomena were also demonstrated on isolated rat aortic rings and isolated fragments of the human tibial arteries obtained from the amputated limbs. TMAO induced an accumulation of mineral content in both ex vivo experiments, leading to upregulation of the genes responsible for osteoblast-like transdifferentiation of VSMC. The same authors analyzed the presence and advancement of aortic calcification by means of CT assessment and histomorphometric analysis in subtotally nephrectomized (CKD) rats injected intraperitoneally with TMAO and fed with high phosphate and high calcium diet. Both techniques of VC assessment revealed that TMAO significantly induces aortic calcification in CKD animals (and that the development of lesions was linked to the increased expression of Runx2 and BMP2 mRNA and proteins). It has also been demonstrated that TMAO increases serum interleukin 1β (IL-1β) in CKD rats (which may suggest that TMAO-induced inflammation may contribute to VC in chronic uremia). In addition, TMAO was shown to activate NLRP3 inflammasome and upregulate NF-κB—both factors critically involved in IL-1β transcription. Since TMAO is a gut-derived toxin synthetized by gut microbiota, it has been attempted to modify TMAO synthesis by using antibiotics to suppress TMAO-generating bacterial strains. Interestingly, although in uremic rats treated with antibiotics serum creatinine remained elevated and comparable with those receiving saline, serum TMAO decreased significantly and this decrease was accompanied by lowered Runx2 and BMP protein expression. Thus, gut microbiota modification—having no influence on the advancement of uremia—almost completely abolished calcium and phosphate accumulation in the aorta. The cited series of elegant experiments demonstrated pivotal role of TMAO in the development of VC and highlighted several mechanisms of its contribution to this process [80]. Other experiments performed earlier also suggested the role of TMAO as a factor triggering vascular inflammation, oxidative stress and endothelial dysfunction, but the study by Zhang et al. demonstrated for the first time such a direct and strong influence of this toxin on VC [81,82].

Yazdekhasti et al. have demonstrated the possible role of TMAO in atherogenesis in apo-E-null mice. In their experiments, authors exposed mice to three diets with an equivalent content of protein, but originating from different sources, namely fish, casein and soy (diets were also comparable in terms of calories, carbohydrate, fat, etc.). Interestingly, diet based on fish-derived protein led to the development of more advanced atherosclerosis in aorta, and the atherosclerotic lesions were also more calcified. Animals fed with fish protein-based diet were characterized with significantly higher mRNA expression of several inflammatory markers (including cluster of differentiation molecule 36 [CD36], IL6 and intercellular adhesion molecule 1 [ICAM-1]) in aortic wall as compared to those fed with casein-based or—especially—soy-based diet. Since TMAO concentration was approximately six times higher in animals receiving fish-based protein, as compared to remaining two diets, it is fair to speculate that TMAO originating from fish protein metabolism may be the key factor responsible for observed differences [83]. One could conclude that feeding those animals with fish-meat based diet was almost as harmful as direct exposition to TMAO injection in other experiments. This is a quite paradoxical observation given the fact that fish are among the products widely recommended as a component of healthy diet (although due to benefits related to fish oil rather than to fish protein). In addition, in a described model the apolipoprotein E deficiency might be an important modifier of an effect of TMAO.

Human studies provide equivocal results. On one hand, observational study in 4007 patients subjected to elective coronary angiography has demonstrated the relationship between fasting plasma levels of TMAO and incident major adverse cardiovascular events (death, myocardial infarction or stroke) during 3 years of follow-up (hazard ratio for highest vs. lowest TMAO quartile equaled 2.54 and remained highly statistically significant after adjustment for traditional cardiovascular risk factors; this trial did not look at the prevalence and advancement of VC) [84]. However, analysis of the Coronary Artery Risk Development in Young Adults (CARDIA) study did not demonstrate the influence of TMAO on the onset or progression of coronary artery calcification score (the direct, CT-based quantification method of calcium-phosphate deposits within coronary arteries), nor common carotid artery intima-media thickness (widely accepted as the sensitive measure of atherosclerosis) over ten-year follow-up [85]. Similar results were obtained in dialysis patients—in most of the studies no association was demonstrated between serum TMAO and all-cause or cardiovascular mortality (although in one of them—namely the Hemodialysis (HEMO) study—separate analysis revealed harm related to high serum TMAO limited to White patients on dialysis, without such effect among Blacks) [79,86,87].

In summary, although experimental data clearly point on the pivotal role of discussed toxins on the development of VC, the clinical studies analyzing an outcome of the patients with different stages of CKD depending on serum levels of uremic toxins provide equivocal results, and—as for now—no firm conclusion on their impact on patient-oriented endpoints can be drawn [78,79,84,85,86,87,88,89,90,91].

**Figure 1:**
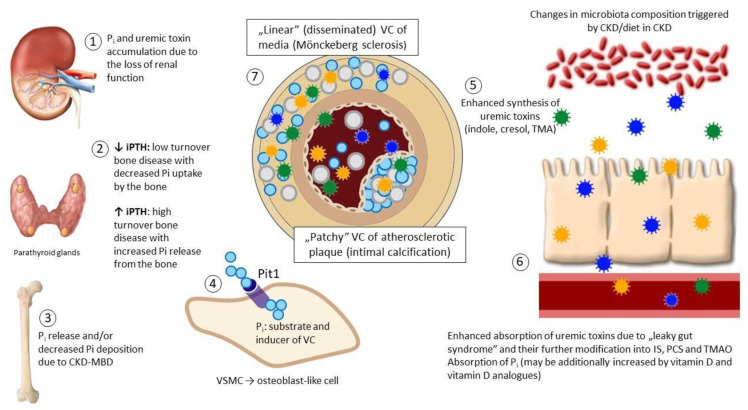
An overview of vascular calcification (VC): The interplay between gut microbiota, kidney, parathyroid glands, bone and the vessels. Loss of nephrons (1) leads to phosphate retention that stimulates the synthesis and release of phosphaturic hormones, including parathyroid hormone (PTH) in parathyroid glands (2). Typically, secondary (renal) hyperparathyroidism develops which results in decreased phosphate reabsorption in proximal renal tubules, but increases bone turnover, promoting phosphate mobilization from the bone (3). Increasing evidence indicates that low-turnover (adynamic) bone disease is even more prevalent scenario in chronic kidney disease (CKD)—for several and not fully understood reasons PTH remains stable or only moderately elevated (“relative” hypoparathyroidism additionally aggravated by the resistance of the bone to PTH) and the bone remodeling slows down (2). In this situation absorbed dietary phosphate is not incorporated into the bone and remains in circulation (3). In both situations excess phosphate serves as a substrate and activator of VC (4). Uremic toxins (indoxyl sulphate (IS), p-cresyl sulphate (PCS) and trimethylamine-*N*-oxide (TMAO)) are normally synthetized by the gut microbiota; such a synthesis becomes excessive due to CKD-driven changes in microbiota composition (5); in addition, toxins accumulate due to the decreased renal clearance and increased gut absorption (6). Uremic toxins modify the phenotype and function of vascular smooth muscle cells (VSMC) and endothelial cells (EC) acting in concert with phosphate and promote VC which becomes excessive and disseminated, involving predominantly media layer beyond atherosclerotic plaque (7). Abbreviations: CKD-MBD—mineral and bone disorders of chronic kidney disease.

The pathways involved in the development and progression of the VC are displayed on Figure 1 and the experimental and clinical data from selected studies—summarized in Table 1.

## 5. Modification of Gut Microbiota to Prevent VC in CKD?

Modification of microbiota composition and metabolism has been postulated for a long time as an attractive way to control uremic toxemia (and possibly—to inhibit such systemic consequences of CKD as VC). Prebiotics, probiotics, modification of diet composition, the use of non-absorbable antibiotics or supplementation of amino acid ketoanalogues may represent strategies to influence the local synthesis and absorption of uremic toxins by means on their impact on microbiota [47,92,93]. Several strategies to decrease phosphate absorption (phosphate binders, the use of compounds that inhibit passive paracellular and active sodium-dependent transcellular phosphate transport in the intestinal epithelium) may additionally limit the availability of this key inducer and substrate for VC [94,95,96]. To date, despite an excellent data from animal experiments, only small studies are available in humans that demonstrated an impact on microbiota manipulation on biochemical variables (including inflammatory markers). The impact of such interventions on clinically meaningful, patient-oriented endpoints has not been shown to date [45]. This statement holds true also when low-phosphate diet or phosphate binders are considered—carefully performed and comprehensive analyses have demonstrated (at best) that one phosphate binder may possibly be better than another in terms of particular side effects or the onset of surrogate endpoints, but no clinically meaningful benefits of any phosphate binder have been documented on such outcomes as: Cardiovascular death, myocardial infarction, stroke, fracture or coronary artery calcification not only when comparing different phosphate binders but also when comparing phosphate binders vs. placebo [97,98].

## 6. Summary and Conclusions

In summary, almost all known pathways contributing to VC in CKD can be linked at some way with the molecular effects of gut microbiota-derived uremic toxins. The promotion of VC starts early in the course of CKD. Theoretically, exposure to these toxins is largely preventable by apparently simple manipulations on gut microbiota. Unfortunately, to date no intervention performed in humans demonstrated efficacy in terms of protection from progression of CKD, nor protection from systemic consequences of CKD (including cardiovascular morbidity). To date none of the interventions applied to treat CKD (and more specifically—CKD-MBD) was demonstrated to stop or reverse VC. Even successful kidney transplantation with at least partial restoration of kidney function does not stop or reverse this process, although is usually able to slow it down [99,100,101,102]. However, due to enormous complexity of mechanisms leading to VC in humans, modification of one contributing pathway (gut-derived toxin synthesis and absorption) would not allow to control VC at the clinical level. Most of the microbiota-modifying interventions would probably not be effective on long term due to adherence and compliance, polypharmacy, tolerance and side effects, costs, etc. Thus—although extremely attractive from the conceptual point of view—in our opinion manipulations on gut microbiota will not become the Holy Grail in VC prevention.

## Figures and Tables

**Table 1:** Uremic toxins: Selected mechanisms of endothelial damage, vascular remodeling and VC. Uremic toxins and cardiovascular (CV) outcome in clinical studies.

IS ^1^		
Model	Design and Methods	Key Findings
In vitro [75]	Medium containing IS at concentrations ranging between 100 and 1000 μmol/L added to cultured VSMC for 24 h or at a concentration of 500 μmol/L added to cultured VSMC for various durations (12–72 h)	Dose- and time-dependent osteoblastic differentiation of VSMC Enhanced calcium deposition within culture Increased expression of Pit-1 mRNA and protein in VSMC Activation of c-Jun N-terminal kinase implicated in IS-induced Pit-1 expression and activation
In vitro [25]	Endothelial progenitor cells cultured with extracelluar matrix vesicles (EMV) derived from IS-treated endothelial cells human umbilical vein endothelial cells (HUVEC) incubated with IS at the concentration of 256 μg/mL to obtain EMV	IS activated oxidative stress, apoptosis and EMV release from humen umbilical vascular endothelial cells (HUVEC) IS activated increased expression of adhesion molecules by HUVEC EMV induced by IS characterized with upregulation of the cluster of miRNA promoting inflammation, apoptosis and cellular senescence EMV derived from IS-treated HUVEC induced oxidative stress and decreased angiogenic potential of endothelial progenitor cells EMV derived from IS-treated HUVEC increased NFκB expression in endothelial progenitor cells
In vitro [27]	HUVEC incubated with IS at the concentration of 250 μmol/L to obtain matrix vesciles (MV) VSMC incubated with MV from IS-treated HUVEC	IS induced senescent phenotype of HUVEC and MV release MV obtained from IS-treated HUVEC induced calcium deposit accumulation in VSMC in culture MV obtained from IS-treated HUVEC induced expression of inflammatory chemokines and cytokines in VSMC in culture
In vitro [28]	EC, VSMC and rat aortic rings exposed to IS at the concentration of 250 μmol/L, phosphate 3 mmol/L or both	Conditioned media from phosphate- and IS-treated EC induced calcium deposition in cultured VSMC Treatment of EC with phosphate and IS increased IL8 mRNA and protein expression in these cells Phosphate and IS induced osteoblastic differentiation of VSMC Phosphate and IS induced vascular calcification in rat aortic rings and increased mRNA expression of rat IL8 homologues in aortic rings Antagonizing IL8 partially prevented calcification in cultured VSMC and rat aortic rings treated with IS
In vitro [68]	Human aortic VSMC incubated with IS at concentrations ranging between 250 and 1000 μmol/L	In cultured VSMC IS downregulates miR-29b, suppressor of the Wnt/β-catenin pathway (Wnt/β-catenin pathway stimulates VC) miR-29b expression decreased and RUNX and osteopontin (OPN; markers of osteoblastic differentiation) increased in human radial artery samples obtained from end-stage kidney disease (ESRD) patients during arterio-venous fistula surgery treated with IS
In vitro and in vivo rat model [71]	Medium containing IS at a concentration of 250 μmol/L added to cultured VSMC IS added to drinking water Serum concentration 13.66 ± 1.19 mg/dL	IS potentiated Ang II-mediated signaling in VSMC IS augmented Ang II-mediated phosphorylation of extracellular signal-regulated kinase (ERK) and epidermal growth factor receptor (EGFR) IS increased ROS production and reactive oxygen species (ROS)-mediated EGFR expression in aortas from uremic rats
In vivo rat model [76]	IS added to drinking water Serum TAC 69.4 ± 5.7 μmol/L	Increased serum glucose and lower expression of glucose transporter 1(GLUT1) in the aorta (prodiabetic milieu) following exposure to IS 10-fold higher aortic calcium content following exposure to IS as compared to control Activation of acute phase, inflammatory and procoagulation pathways implicated in IS-induced aortic calcification
Clinical [88]	Observational study; analysis of outcome in a cohort of 1273 ESRD patients treated with hemodialysis, depending on the baseline serum IS level (sub-study of the longitudinal HEMO study) Mean baseline serum IS: 2.5 ± 1.2 mg/dL (median 2.4 mg/dL)	No association between serum IS and any of the analyzed outcomes, including: Cardiac death, sudden cardiac death, first CV event, death from any cause
**PCS ^1^**		
**Model**	**Design** **and Methods**	**Key findings**
In vivo rat model [76]	PCS added to drinking water Time-averaged concentration 151.0 ± 40.7 μmol/L	Increased serum glucose and lower expression of GLUT1 in the aorta (prodiabetic milieu) following exposure to PCS 10-fold higher aortic calcium content following exposure to PCS as compared to control Activation of acute phase, inflammatory and procoagulation pathways as implicated in PCS-induced aortic calcification
In vitro [26]	HUVEC incubated with PCS at a concentration of 25 μg/mL to obtain EMV Endothelial cells cultured with EMV derived from PCS-treated endothelial cells	PCS induced EMV release from HUVEC EMV induced by PCS characterized with changes in miRNA profile towards promotion of cellular senescence and reduction in proliferative and angiogenic capacity of endothelial cells EMV derived from PCS-treated HUVEC decreased endothelial cell migration and induced cell senescence
In vitro, in vivo [78]	HUVEC and HVSMC incubated with PCS at a concentration ranging between 0.02 and 0.5 mmol/L Vascular contractility of mouse aortic rings tested with PCS at a concentration of 0.15 mmol/L	PCS activated oxidative stress in endothelial cells (HUVEC) and VSMC (maximum effect at a concentration of 0.15 mmol/L) PCS promoted phenylephrine-induced contraction of smooth muscles within aortic wall PCS promoted aortic wall remodeling
Clinical [88]	Observational study; analysis of outcome in a cohort of 1273 ESRD patients treated with hemodialysis, depending on the baseline serum PCS level (substudy of the longitudinal HEMO study) Mean baseline serum PCS: 3.3 ± 1.7 mg/dL (median 3.3 mg/dL)	No association between serum PCS and any of the analyzed outcomes, including: Cardiac death, sudden cardiac death, first CV event, death from any cause
Clinical [89,90] Same study, two publications	Observational study; analysis of outcome in a cohort of 175 ESRD patients treated with hemodialysis, depending on the baseline serum PCS level Mean baseline total serum PCS: 19.0 ± 0.9 mg/L (median 18.9 mg/L) Mean baseline free serum PCS: 2.59 ± 0.17 mg/L (median 1.97 mg/L)	Baseline concentration of free but not total PCS predicted all-cause mortality over median follow-up period of 30 months Baseline concentration of free but not total PCS predicted new CV event defined as composite of death from cardiac causes, non-fatal myocardial infarction, myocardial ischemia, ischemic stroke or new peripheral vascular disease Free serum PCS concentration predicted new CV events in the whole cohort and in non-diabetic, but not in diabetic patients
**TMAO ^1^**		
**Model**	**Design and Methods**	**Key findings**
In vitro, ex vivo, in vivo, clinical [50]	Rat aortic VSMC incubated with TMAO at concentrations of 100, 300 and 600μmol/L Human VSMC incubated with TMAO at concentrations of 100, 300 and 600μmol/L Rat aortic rings incubated with TMAO at concentrations of 100 and 300 μmol/L Human tibial arterial rings (obtained from amputated limbs) incubated with TMAO at concentrations of 100 and 300 μmol/LRats treated with TMAO injected intraperitoneally (3.3 μmol/kg daily) (serum TMAO level between 23 and 35μmol/L; exact values not provided in the text, deduced from the figure) CKD patients with serum TMAO concentration measured and VC assessed using coronary artery calcification (CAC) (serum TMAO level ranging between 40 and 200 μmol/L; exact values not provided in the text, deduced from the figure) Effect of gut decontamination on vascular lesions in experimental animals	TMAO induced mRNA expression of Runx2 and BMP2 in cultured rat and human VSMC, isolated rat aortic rings and isolated fragments of human tibial arteries TMAO induced VC in isolated rat aortic rings and isolated fragments of human tibial arteries TMAO injected intraperitoneally increased VC in subtotally nephrectomized (CKD) by means of CT assessment and histomorphometric analysis TMAO increased serum IL-1β in CKD rats TMAO activated NLRP3 inflammasome and upegulated NF-κB Patients with aortic arch calcification (AAC) were characterized with significantly higher serum TMAO level as compared with those without AAC; the advancement of AAC correlated with serum TMAO Gut decontamination with antibiotics led to significantly decreased serum TMAO and lowered Runx2 and BMP protein expression in CKD animals, and almost completely abolished calcium and phosphate accumulation in their aortas (otherwise having no influence on the development of CKD)
In vivo [83]	apo-E-null mice fed with diets with an equivalent content of protein, but originating from fish, casein or soy (identical in terms of calories, carbohydrate, fat, etc.) TMA content in experimental diets: 529 mg/kg (fish), 24.5 mg/kg (casein) and 23 mg/kg (soy)	Serum TMAO concentrations in animals fed with experimental diets: 7.0 μmol/L (fish), 1.0 μmol/L (casein) and 1.5 μmol/L (soy) More advanced atherosclerosis and more calcified atherosclerotic lesions in animals fed with fish protein Aortic wall mRNA expression of of several inflammatory markers (including CD36, IL6 and ICAM-1) significantly higher in animals fed with fish protein
Clinical trial [85]	Observational trial of 817 apparently healthy participants aged between 33 and 55 years with serial measurements of CAC and common carotid artery intima-media thickness (CCA-IMT) Baseline median TMAO concentration of 2.6 μmol/L (interquartile range: 1.8–4.2)	No association between baseline serum TMAO concentration and progression in CAC nor CCA-IMT value; no association between baseline serum TMAO concentration and CV events nor GFR loss (10-year follow-up)
Clinical [84]	4007 patients who underwent coronary angiography and had baseline serum TMAO assessment Median baseline serum TMAO level 3.7 (interquartile range 2.4–6.2) μmol/L	Baseline serum TMAO level significantly associated with the risk of major adverse cardiovascular events (defined as: death, MI or stroke) in three-year follow-up
Clinical [79]	235 ESRD patients treated with hemodialysis, with baseline serum TMAO assessment Mean baseline serum TMAO 50 ± 32 μmol/L (median 43 μmol/L)	No association between baseline serum TMAO and all cause death, CV death or CV hospitalization (median follow-up of 4 years)
Clinical [86]	1242 patients treated with hemodialysis, with baseline serum TMAO assessment from the EVOLVE prospective randomized trial Baseline serum TMAO range: 2.5–1103.1 μmol/L (considered “extraordinarily wide range” by authors)	No association between baseline serum TMAO, all-cause death and vascular composite outcome defined as: Cardiovascular death, MI, peripheral vascular event, stroke and hospitalization for unstable angina
Clinical [91]	Observational study; analysis of outcome in a cohort of 1273 ESRD patients treated with hemodialysis, depending on the baseline serum TMAO level (sub-study of the longitudinal HEMO study) Mean baseline serum TMAO: 101.9 ± 63.9 μmol/L (median 88 μmol/L)	No association between baseline serum TMAO and any of the analyzed outcomes, including: Cardiac death, sudden cardiac death, first CV event, death from any cause for the whole group and among Black patients TMAO significantly associated with all above endpoints among Whites The difference in outcome between races despite comparable serum TMAO between respective groups

^1^ IS, pCS and TMAO concentrations in experiments comparable to average serum concentrations in uremic patients.

## Data Availability

Not applicable.
